# Nanopore Impedance Spectroscopy Reveals Electrical Properties of Single Nanoparticles for Detecting and Identifying Pathogenic Viruses

**DOI:** 10.1021/acsomega.3c00628

**Published:** 2023-04-06

**Authors:** Kazuki Kitta, Maami Sakamoto, Kei Hayakawa, Akira Nukazuka, Kazuhiko Kano, Takatoki Yamamoto

**Affiliations:** † Mechanical Engineering, Tokyo Institute of Technology, Ishikawadai 1-314, 2-12-1 Ookayama, Meguro-ku, Tokyo 152-8550, Japan; ‡ Material Research and Innovation Division, DENSO CORPORATION, 1-1 Showa-cho, Kariya, Aichi 448-8661, Japan

## Abstract

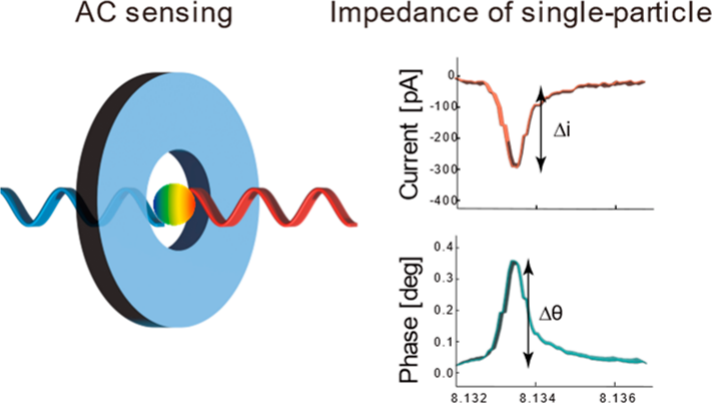

In the conventional nanopore method, direct current (DC) is used to study molecules and nanoparticles; however, it cannot easily discriminate between materials with similarly sized particles. Herein, we developed an alternating current (AC)-based nanopore method to measure the impedance of a single nanoparticle and distinguish between particles of the same size based on their material characteristics. We demonstrated the performance of this method using impedance measurements to determine the size and frequency characteristics of various particles, ranging in diameter from 200 nm to 1 μm. Furthermore, the alternating current method exhibited high accuracy for biosensing applications, identifying viruses with over 85% accuracy using single-particle measurement and machine learning. Therefore, this novel nanopore method is useful for applications in materials science, biology, and medicine.

## Introduction

The nanopore method measures changes in an electric current when a measurement target passes through a nanometer-sized pore. Although most conventional experimental methods provide time- and population-averaged images, nanopore measurement allows for the investigation of how individual particles behave and interact with each other. The nanopore method can reveal structural and spatial dynamics on a single-particle level at the sub-millisecond scale, making it an important tool for exploring dynamic processes in life sciences, chemistry, medicine, and other fields.^1,2^ Nanopores have been actively studied since the late 1990s, mainly for applications in DNA sequencing.^3−8^ Recently, nanopore sensing has expanded beyond this field to various applications, including DNA–protein interaction research,^9,10^ biomolecular sensing,^11−14^ biological screening and diagnostics,^15^ protein sequencing,^16^ and detection of bionanoparticles such as viruses^17−20^ and extracellular vesicles.^21,22^

The nanopore method often yields a poor signal-to-noise ratio (SNR) because it measures low-intensity signals over a short time period.^23^ Extraction of weak signals from a large field of background noise is a key issue in nanopore measurement.^24^ Recent research on low-noise broadband measurement circuits has facilitated analyses at the microsecond to sub-microsecond scale, thereby leading to the improvement of the sensitivity and accuracy of the nanopore method.^25−28^ However, the conventional nanopore method is based on direct current (DC) measurements, and the available output information is limited to changes in the magnitude and duration of currents that exhibit a pulse-like response.^29^ By contrast, the application of an alternating current (AC) for measurements offers the advantage of evaluating material characteristics such as resistivity, permeability, and permittivity in principle.^30−32^

AC applications, including DNA impedance sensing,^33^ cancer diagnosis,^34^ pathogens,^35^ and low molecular weight compounds,^36^ have been reported, taking advantage of both the large surface area and mass transport properties of nanoporous membranes and nanopore arrays. Furthermore, there are examples of a single nanopore measuring AC in single particles or molecules, such as in studies of blocking events in channel proteins^37^ or DNA–protein interactions.^38^ However, the use of AC has been limited to current value changes. There has not yet been a report determining the impedance or capacitance of a single particle from simultaneous measurements of current value and phase.

In this preliminary study, we aimed to demonstrate an AC measurement-based nanopore method to realize the multimodal characterization of a single nanoparticle. Using this method, we first demonstrated single nanoparticle detection. Next, the frequency characteristics of a single particle were measured to evaluate the performance of this method in terms of sensing applications, which distinguish particles of the same size based on differences in surface condition and material properties. We also investigated virus detection as an application of multimodal AC-based measurement of single nanoparticles. Furthermore, we employed a machine learning technique to improve identification performance. The results showed that the multimodal measurement capability improves the identification performance when machine learning techniques are employed to identify the virus type.

## Results and Discussion

**Figure 1:**
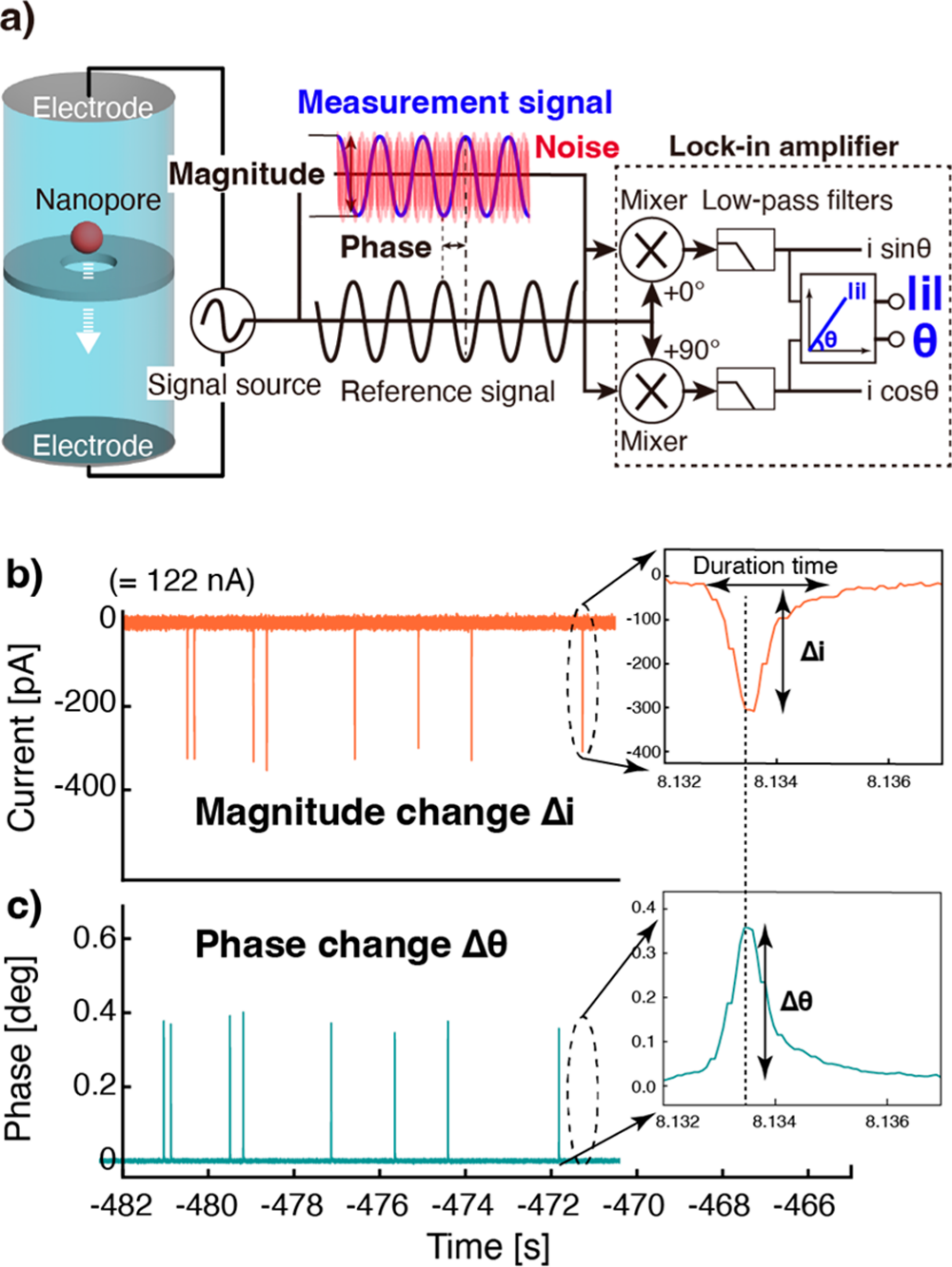
Lock-in measurement system for AC nanopore measurement. a) Overview of the measurement system. The time traces show typical measurement signals in b) the magnitude changes of the current (Δ*i*) and c) the corresponding phase changes (Δθ) of the current. A pulse waveform is obtained because the nanopore (NP100) is momentarily blocked by individual 100 nm polystyrene particles. The inset shows a magnified view of the magnitude (Δ*i*) and phase change (Δθ) of the same single-particle-derived current pulse, which is used to characterize the individual particles flowing through the pores.

Figure 1a shows the system configuration of the AC nanopore method using a lock-in method. The lock-in method is a technique used to extract weak signals from a noisy background via phase-sensitive homodyne detection by applying a specific frequency modulation to the sample.^39^ Briefly, the measurement signal is split into two identical frequency signals: one used for the measurement, in which particle information and various noises are overlaid, and the other for the reference signal, which is multiplied by the measured signal to extract only the signal components of the same reference signal frequency. Consecutively, the magnitude and phase of the current can be measured. Because the lock-in amplifier converts the measured AC signal to a DC signal as a root-mean-square value (RMS), a current waveform similar to that obtained by the DC nanopore method can be obtained (Figure 1b,c, Supporting Movie 1). When a particle passes through the nanopore, the current is blocked. This synchronously leads to downward pulsing to decrease the current magnitude and upward pulsing to increase the current phase. The phase of the current is more advanced than the voltage, indicating a capacitive response.^40^ Here, the baseline current and phase represent responses of the nanopore itself, which exhibits a capacitive impedance. This clearly indicates that the pulse signal originates from a particle; however, it also suggests that the characteristics of the nanopore chip must also be considered with regard to quantitativeness. The current magnitude and phase changes always occur synchronously, indicating that they originated from a single particle (Figure 1b,c inset, Supporting Movie 2). The nanopore used herein has a conical shape with a wider diameter at the exit than at the entrance, resulting in gradual tailing on the exit side (right side) of the waveform.^20,41^ The rectifying effect of conical nanopores may cause a bias in the ionic current due to electroosmotic flow under an AC electric field.^42,43^ Herein, however, no clear rectification effect appeared, likely owing to the low applied voltage, and there was no DC bias in the baseline of the measurement signal or distortion of the waveform (Figure S1).

We next evaluated the frequency dependence of the magnitude and phase displacements of the current. The influence of the material and surface conditions of the particles on the frequency characteristics was also evaluated. Because the nanopores used were mounted on a measurement jig and stretched to control the aperture size, the reproducibility of the aperture size is not high. Therefore, a series of measurements were all performed using the same nanopore under the same stretching conditions, except that the particles were different.

**Figure 2:**
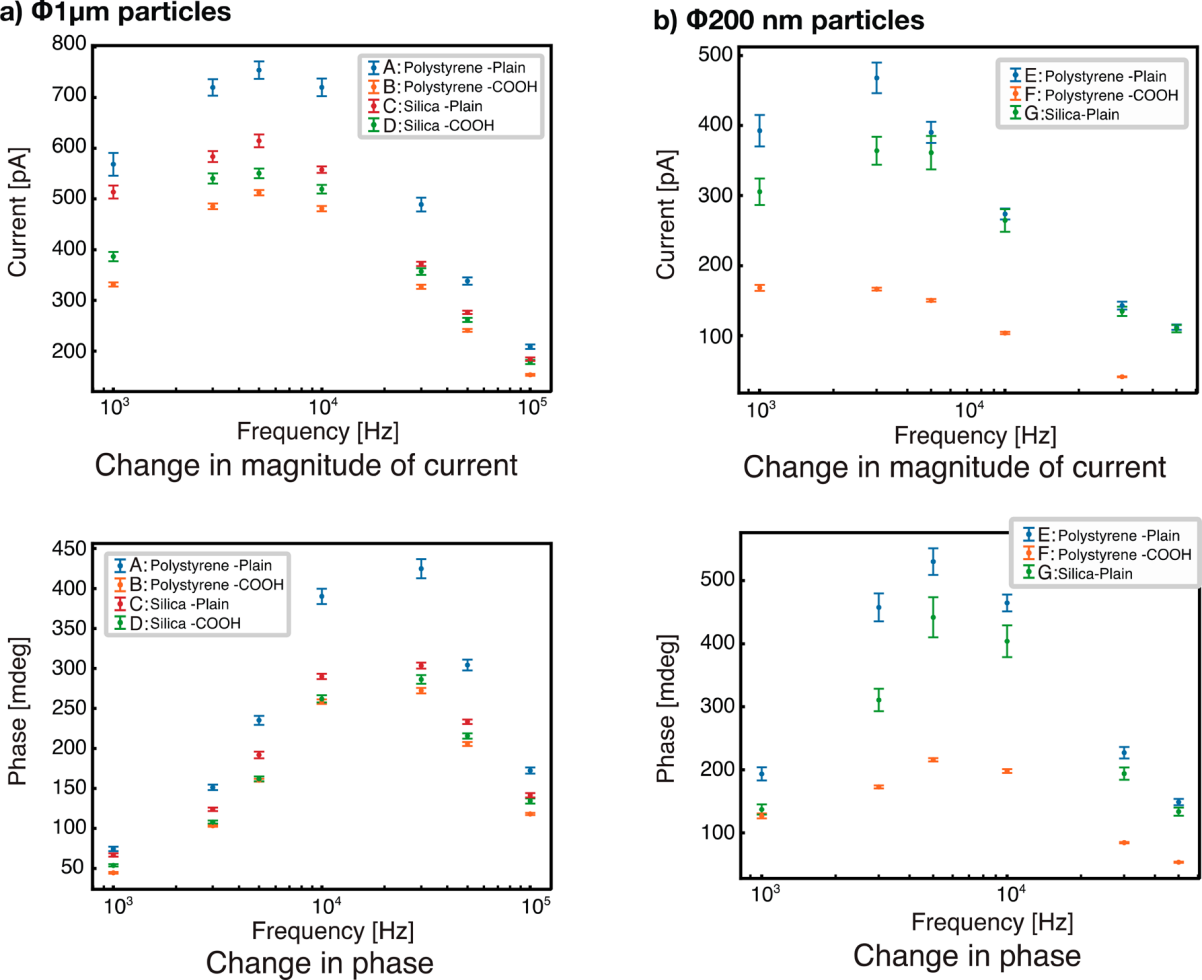
Frequency response of nanoparticles. a) and b) Current and phase changes of 1 μm and 200 nm particles, respectively. For the 200 nm particles, only phases above 50 kHz were detected. Each point represents the mean of at least 200 measurements and error bars represent standard errors.

Figure 2a,b shows the frequency characteristics of the magnitude and phase changes of the current for particles of 1 μm and 200 nm, respectively. The types of particles used are shown in the insets of Figure 2b,c, which have the same size, but different materials and surface modifications (Table S1). The obtained frequency characteristics were different for each particle, despite the same particle diameters. Particles of the same size and material, but with different surface modifications were compared: particles with 1 μm diameters, A (polystyrene, no modification, plain) and B (polystyrene, COOH), were compared against C (silica, plain) and D (silica, COOH), and particles with 200 nm diameters, E (polystyrene, plain) and F (polystyrene, COOH), were compared against G (silica, COOH). As shown in Figure S2, the particles are identical in size and shape at a level that cannot be determined using the scanning electron microscope image.

Both the magnitude and phase of the current were larger for the plain particles. This suggests that the impedance of the particles was affected by the surface conditions. Differences in impedance were also observed when different combinations of materials with the same size and surface modification were compared: In this case, samples A and B were compared against C and D, and samples E and F were compared against G. Despite having the same size and surface condition, it was found that particles of different materials could be distinguished.

Although the surface charge density of polystyrene plain particles was unknown in this measurement, the degree of change was greater for plain particles (7 μmol/g) and COOH particles (2 μmol/g), which have high charge densities under neutral pH conditions, than for silica particles. Polarization phenomena such as concentration polarization^44^ and counterion polarization^45−47^ may be involved in causing the surface-charge-dependent current variation. However, the surface charge and electric field strength used in this study are very small compared to the surface charge and electric field strength of particles with pronounced concentration polarization.^44^ Therefore, it is inferred that the effect of counterion polarization, in which an external electric field displaces the counterions and causes polarization, is more notable.

However, particles B (COOH polystyrene) and D (COOH silica) with the same surface potential (2 μmol/g) showed lower current values for polystyrene particles than for silica particles, suggesting that not only counterion polarization but also various polarization phenomena may act simultaneously. Although a more detailed evaluation is needed in the future, in principle, it is possible that various polarization phenomena may act simultaneously, depending on the frequency.^48−50^

When focusing on a certain frequency, it is expected that the accuracy of particle size measurement is not high when determining the particle size from current values because current values differ for particles of the same size but with different surface conditions and materials. However, when current values and particle diameters were compared between particles from the same manufacturer with the same surface (COOH) and material (polystyrene), but differing only in particle size, the current values were found to be proportional to particle volume, as in the conventional DC nanopore method. A histogram of the particle size distribution for traceable particles of the same surface modification and materials with high particle size accuracy is shown in Figure S3. In this measurement, both particle size accuracy within 2% and coefficient of variation within 5% were obtained.

**Figure 3:**
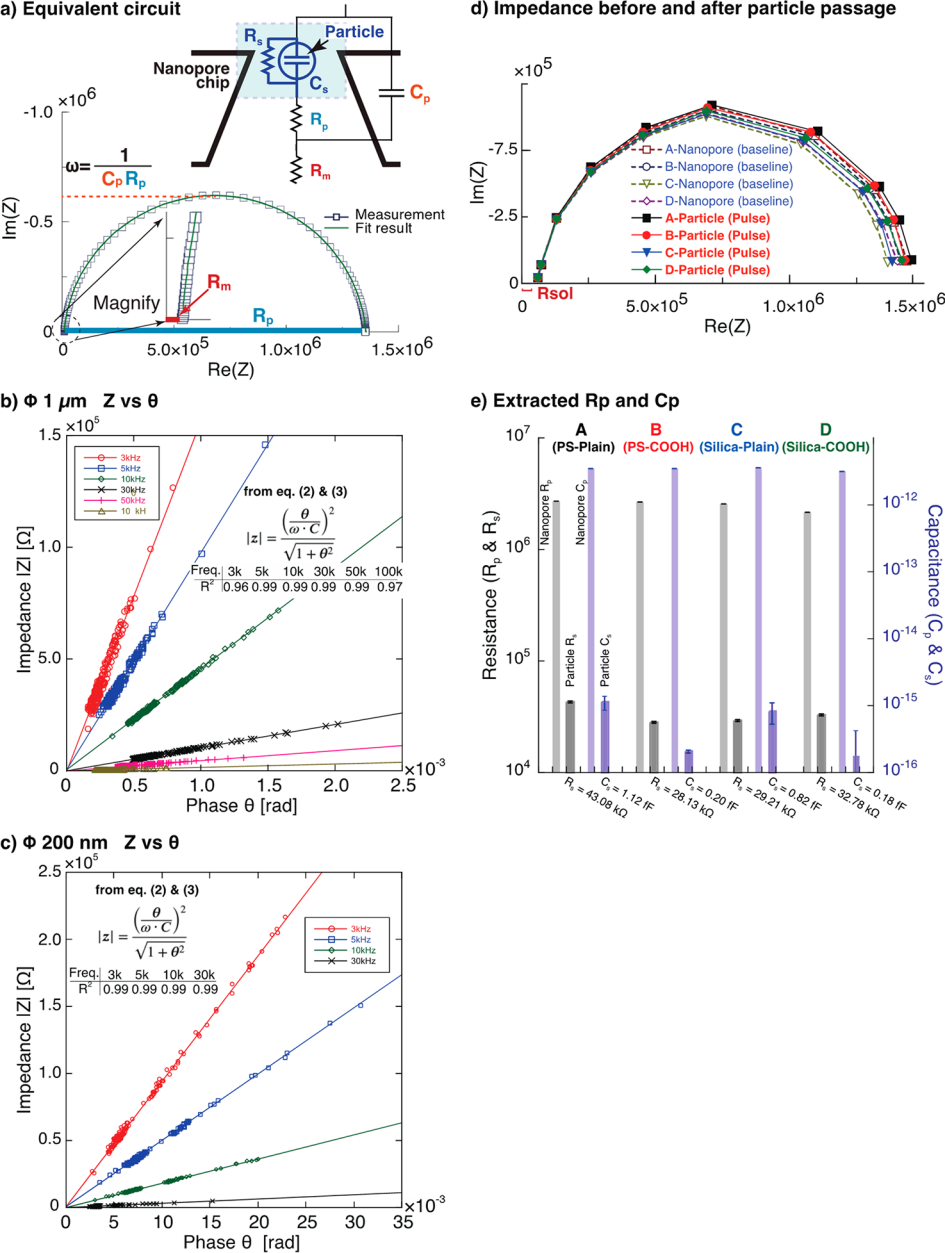
Frequency response of impedance *Z* versus phase change θ and the evaluated resistance and capacitance derived from single particles. a) Nyquist plot of the frequency response of a nanopore chip (NP400) for 1 μm particle measurement and the electrical equivalent circuit of the nanopore chip and particle-derived impedance changes. The colors of the letters indicate the correspondence between the Nyquist plot and the equivalent circuit elements. The highlighted areas in light blue correspond to the impedance change due to the particle corresponding to the current pulse obtained by the measurement. Particle-size-dependent frequency response of *Z* versus θ plots for b) particles of 1 μm in diameter and c) particles of 200 nm in diameter. In the figure, the fitting equations derived from eqs 2 and 3, and the fitting *R*^2^ values at each frequency are shown. d) Nyquist plot showing the frequency response of the impedance before and after particle passage. The impedance before the passage is the impedance of the nanopore chip alone, and the impedance after the passage is the impedance obtained from each particle pulse. e) Resistances *R*_s_ and capacitances *C*_s_ in the impedance change are derived from the particle origin calculated from panel d.

For further quantitative evaluation, the particle-derived resistance and capacitance changes were extracted by subtracting the impedance of the nanopore chip from the particle-derived impedance. Figure 3a shows the electrical equivalent circuit of the measurement system of the AC nanopore measurements. The impedance of the nanopore chip was modeled as a parallel circuit of resistance *R*_*p*_ and electrical capacitance *C*_*p*_ with solution resistance *R*_*m*_ connected in series. The highlighted area in light blue corresponds to the change due to particles, which corresponds to the current pulse obtained by the measurement. The change in admittance (*Y*_*s*_) and phase (θ_*s*_) owing to the presence of particles was modeled as a parallel circuit of resistance *R*_*s*_ and electrical capacitance *C*_*s*_. Because the measured θ_*s*_ was very small, it was approximated using eq 3.
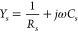
1
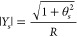
2
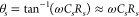
3

From these equations, the changes in resistance and capacitance owing to the passage of particles were determined. An example of fitting this equivalent circuit to the Nyquist plot of a nanopore chip by the complex nonlinear least-squares method is shown by the green line in Figure 3a. The goodness of fit by weighted sum of squares was less than 4 × 10^–6^ for all fittings, and the estimation error for each element was less than 1%, indicating high agreement between the measurements and the equivalent circuit.

Figure 3b,c shows the frequency dependence of the correlation between the impedance *Z* = 1/*Y* and phase change θ with regard to the particle sizes of COOH-modified polystyrene particles with diameters of 1 μm and 200 nm, respectively. Although highly monodisperse particles were used, the actual particle size varied by several percent, resulting in the distribution of current magnitude and phase according to particle size, with large particles distributed diagonally in the upper right and small particles in the lower left in Figure 3b,c. A high linearity was obtained in the slope at all frequencies in the measurement range, and the slope increased as the frequency increased. The high *R*^2^ values obtained by least-squares fitting based on eqs 2 and 3 demonstrate the validity of eqs 1–3 as the equivalent circuits.

To reduce errors due to particle size variation, the measured impedances were averaged over multiple points across the entire measurement frequency range and approximated using the least-squares method to extract the portion of the impedance change derived from the particles. Only the 1 μm diameter group was analyzed because the 200 nm group had a large measurement error. Figure 3d is a Nyquist plot showing the impedance characteristics of the nanopore chips obtained from the baseline and those of the particles passing through the nanopore chips obtained from the current pulse for each of the four types of particles of 1 μm in diameter used in Figure 2. Figure 3e shows the change in resistance and capacitance derived from each of the four types of particles. Depending on the type of particle, the variation in resistance is 28–43 kΩ and that in capacitance is 0.2–1 fF, clearly different values. Regarding the capacitance, because the relative permittivities of water and polystyrene at 20 °C are approximately 80 and 2–3, respectively, a decrease in capacitance with particle volume was expected, but in fact the capacitance increased, probably because the effects of counterion polarization^45−47^ were more dominant than those from the permittivity of the material. However, because the capacitance value is near the detection limit of the measuring instrument and includes errors in equivalent circuit fitting, a careful discussion and quantitative analysis that takes into account the accuracy of the entire measurement system, along with further improvements in measurement performance, are needed to determine the origin of the electric capacitance. Nevertheless, these results suggest the possibility of a method that measures *R* and *C* derived from single nanoparticles, thereby determining the dielectric constant.

We evaluated the performance of the AC nanopore method as a sensor in the detection and classification of microorganisms, such as viruses, based on its high sensitivity to material properties. To achieve highly accurate classification from noisy nanopore measurement data, machine learning-assisted classification was performed.^22,51−53^ The random forest method was used as the machine learning algorithm,^54^ and current and phase change waveforms were used as training data. Random forests combine the outputs of an ensemble of regression trees to predict the value of the response variable, reducing the risk of overfitting and enabling robust classification against outliers and noise.^52^

**Figure 4:**
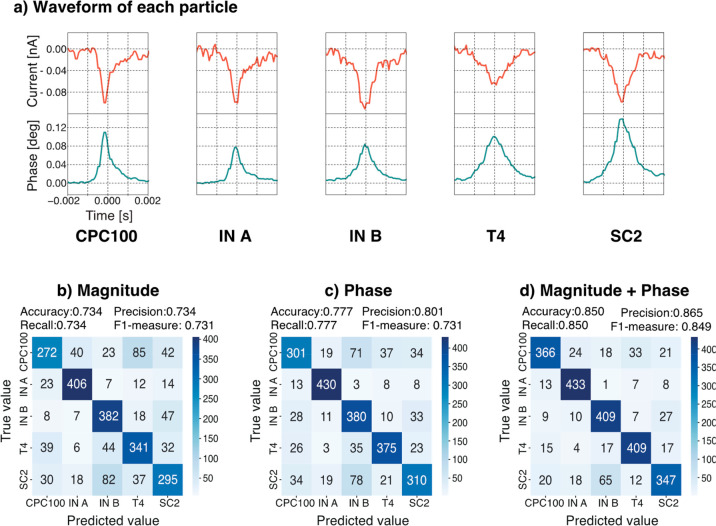
Representative current magnitude and phase shift waveforms and confusion matrix resulting from machine learning classification of four viruses and one spherical particle using the random forest method, where CPC100 is the particle with a 100 nm diameter; IN A and IN B are influenza virus types A and B, respectively; T4 is T4 phage; and SC2 is severe acute respiratory syndrome coronavirus 2 virus. A comparison was made between the cases of using only a) the representative current magnitude and phase shift waveforms for each particle. Both the vertical and horizontal axes of each waveform are all on the same scale. Determination using b) magnitude of current, c) phase information alone, and d) both magnitude and phase information. The accuracy, precision, recall, and F1-measure for each are shown. The number in the figure is the count of classified waveforms: The higher the number and the darker the color of the diagonal of the right downward diagonal, the greater the percentage of correct answers.

In a pioneering virus classification study combining the DC nanopore method and machine learning, Taniguchi et al. reported that F1-measure of ≥76% and ≥69%, which are the harmonic means of precision and recall, were achieved for two and three subtypes of coronavirus, respectively, based on current waveforms alone.^53^ While the DC nanopore method distinguishes based on current magnitude information only, the AC nanopore method allows particle identification based on two waveforms, the current magnitude and phase, which corresponds to each viral particle, as shown in Figure 4a. Using the five types of particles, which consisted of four viruses including the influenza subtype and virus-sized artificial particles, as shown in Figure 4a, we first obtained F1 measure of approximately 74% and 77% for classification based solely on the magnitude or phase of the current, respectively, as shown in Figure 4b,c.

Although a direct comparison cannot be made because of the different viruses used, the classification accuracy based on either magnitude or phase of the current was not significantly different between the DC and AC methods. By contrast, when both magnitude and phase were used simultaneously, the accuracy of the AC method improved to approximately 85%. The precision and F1-measure were also approximately 87% and 85%, respectively, as shown in Figure 4d, indicating that the AC nanopore method was more accurate than the DC method in this case. These results suggest that multimodal data collection enabled by the AC nanopore method improved the accuracy of machine learning-assisted virus identification.

## Conclusions

In summary, we demonstrated an AC nanopore method with lock-in measurement to overcome the conflicting pitfalls of ultralow current and broadband in conventional nanopore sensing. We also demonstrated a potential application of this method for virus sensing. To the best of our knowledge, this represents the first example of AC impedance measurement of a single nanoparticle using nanopores.

Although challenges remain in terms of accuracy, we showed a method to measure the resistance and capacitance of a single particle based on changes in the magnitude and phase of the current. Particularly, the AC nanopore method is a practical and widely applicable measurement method owing to its ability to measure electrical capacitance, which is difficult to measure with the conventional DC nanopore method. The ability to distinguish particles by material properties such as permittivity is valuable for not only materials science but also sensing applications. In the case of virus sensing, for example, it is necessary to distinguish various subtypes of the same virus with different sizes and slightly different surface-localized glycans and proteins.^55^ Our results show that the AC nanopore method can distinguish subtypes of viruses based on material properties, including surface conditions, demonstrating the advantage of this method. We believe these findings will lead to advancements in biosensing, particularly of pathological testing methods. Conventional pathogen diagnostic methods, such as immunochromatography and polymerase chain reaction (PCR) testing, are highly specific, but require labeling reagents (such as antibodies, nucleic acids, and sugar chains) that bind to specific microorganisms, thereby necessitating the development of many labeling reagents corresponding to each type of microorganism. Furthermore, these reagents can only be used once. By contrast, the nanopore method has the advantage of not requiring labeling reagents and may be used for long-term continuous monitoring applications. Although the AC nanopore method will likely not be able to compete with PCR in terms of the detection specificity, its practicality makes it useful for primary screening. Beyond the detection of infectious diseases, the AC nanopore method has the potential to be widely employed in various biosensing applications, such as early detection of cancer via extracellular vesicle screening and detecting toxic particulate matter. With further improvements in the sensitivity and accuracy, we believe that the AC nanopore method will contribute immensely to pathological diagnosis and the detection of pathogenic viruses and hazardous particulate matter in the environment, aiding the development of new pandemic countermeasures.

## Methods

### Nanopores

Commercially available NP100 and NP400 urethane nanopores (Izon Science Ltd., Christchurch, New Zealand) were used in this study to measure different particle sizes. The theoretical treatments for resistance change and pore diameter tuning were set based on previous publications.^20,56^ The nanopores were attached to the stretching jaws of the QNano instrument (Izon Science Ltd.), a commercially available nanopore measurement device. We used only the measurement cell and stretching jaws and not the internal measurement circuit, control, or analysis software. The exact size and shape of the nanopores are unknown^20,56^ because the nanopores are irregularly conical in shape and their diameters vary with the degree of stretching. During the measurement, all measurements were performed with the same nanopores set to the same stretch level to eliminate measurement error due to variations in pore size. Because elastomeric materials such as urethane are known to undergo stress softening, such as that as a result of the Mullins effect, during stretching,^57^ measurements were performed after approximately 5–10 stretch cycles to avoid nanopore deformation.^58^

### Measurement Solution

Particles were dispersed in a measurement solution comprising phosphate-buffered saline (17–516Q, Lonza, Morrisville, NC) and an artificial phospholipid-type surfactant (Lipidure BL206, NOF Co., Tokyo, Japan) at a final concentration of 0.01% as an antiadsorption agent. In all experiments, measurements were performed at pH 7.4 and a conductivity of 1.7 S/m.

### Nanoparticles

The three particle types in the 200 nm diameter group included unmodified (plain; 01–00–202), carboxylated polystyrene (01–02–202), and carboxylated silica particles (43–02–202). The surface charge density of the 200 nm particles is 4 μmol/g for the COOH polystyrene particles and that of the 200 nm silica particles is 1 μmol/g. The particles in the 1 μm diameter group included unmodified (plain) polystyrene (01–00–103), COOH-modified polystyrene (01–02–103), unmodified (plain) silica (43–00–103), and COOH-modified silica (43–02–103). The surface charge density of both 1 μm COOH-modified polystyrene and silica particles is 2 μmol/g, while that of the plain silica particles is 7 μmol/g. All particle types were purchased from Micromod Partikeltechnologie GmbH (Rostock, Germany). For all these particles, the manufacturer indicated the particle size, particle concentration, and the surface charge.

### Virus Sample Preparation

*Escherichia coli* (JCM 20135, RIKEN BioResource Center, Kyoto, Japan) at an optical density of 0.5–600 nm was suspended in Luria broth and mixed with *E. coli* phage T4 (NBRC20004, National Institute of Technology and Evaluation, Osaka, Japan) concentrate (10^10^ particles/mL) until a final concentration of 10^6^ particles/mL was reached. The solution was incubated at 37 °C for 24 h and centrifuged at 5000 × *g* for 2 min; the T4 phages in the supernatant were then collected. Thereafter, the culture medium was replaced with the assay medium by centrifugation at 4000 × *g* for 5 min using VIVASPIN 500 (VS0171, Sartorius, Tokyo, Japan) and 14000 × *g* for 5 min using 0.5 mL of Amicon Ultra (C82301, Merck & Co., Rahway, NJ, USA). The concentration of T4 phages was measured using the QNano instrument (Izon Science Ltd.). All other virus particles were purchased and used in the inactivated form. Thimerosal and beta propiolactone inactivation-treated influenza A/Panama/2007/99 (H3N2; 8IN74–1) and Influenza B/Malaysia/2506/04 (8IN75–4) particles were acquired from HyTest Ltd. (Turku, Finland). Heat-inactivated SARS-CoV-2 (VR1986HKTM) was obtained from the American Type Culture Collection (Manassas, VA). Sample pretreatment was performed as that for the T4 phages.

### Lock-In Measurement

A commercial lock-in amplifier (MFIA 5M, Zurich Instruments, Zurich, Switzerland) was used in this study. The measurement voltage and frequency were set at an arbitrary value obtained from the lock-in amplifier, and the current passing through the nanopore was converted to a voltage by a transimpedance preamplifier before input to the lock-in amplifier. A transimpedance amplifier (CA-653F2, NF Corp., Kanagawa, Japan) with a bandwidth of 1 MHz and a gain of 10^6^ V/A was used.

The sampling rate was 60 MHz regardless of the measurement frequency. The low-pass filter (LPF) of the lock-in amplifier was set as third-order, and the bandwidth was 2 kHz. The entire setup, except for the lock-in amplifier, was placed in a shielded box to reduce noise. To further reduce noise due to input capacitance, only coaxial cables (RG-63u, Fujikura Ltd., Tokyo, Japan) with a characteristic impedance of 125 Ω and a capacitance of 33 pF/m were used. To prevent noise from the power supply, the preamplifier was powered by batteries (Eneloop, Panasonic, Osaka, Japan) stored in a shielded box. By taking these various noise countermeasures, the baseline current noise was reduced to 4 pA_pp_ (approximately 1.4 pA_rms_) at 10 kHz under our measurement conditions. It is currently possible to separate noise from signal if the SNR is greater than 2; we performed measurements with an SNR of at least 5 throughout this study.

### Measurement Operation

Unless otherwise specified in the text, the particle suspension was added to the upper cell at a pressure head of 3 mm (30 Pa) in all experiments, and the particle suspension was driven by hydrostatic pressure. A minimum of 200 counts were recorded for all measurements, and a minimum of 500 counts were recorded for machine learning in particular. When measuring multiple particle types in the 1 μm and 200 nm diameter groups, a single nanopore chip and aperture setting was used for each group to ensure that the measurement conditions were identical. The frequency response of the nanopore chip was measured with and without particle measurement solution before and after the measurement to confirm that the impedance of the nanopore chip did not change during the measurement. Each measurement was performed within 10 min to avoid the effect of pressure fluctuations due to decreasing solution volume and changes in conductivity due to evaporation.

### Measurement and Initial Data Processing

LabOne (Release 22.02, Zurich Instruments) software was used to control the lock-in amplifier and record data. Data analyses, including trend correction, separation of signal pulses from the noise floor, pulse counting, and calculation of pulse amplitude and width, were performed using the commercially available data analysis software DIAdem 2021 (National Instruments, Austin, TX) and Python (Ver. 3.7.9). The following libraries were used for the series of analyses: NumPy (basic scientific computing), matplotlib (graphing), SciPy (for advanced scientific computing such as pulse detection and pulse width analysis), pandas (data analysis support), and npTDMS (for large data analysis used for TDMS files, which are binary files of large data used by the product line of National Instruments and can be read on other platforms.)

The basic signal processing flow is as follows: Because LabOne data were outputted in a proprietary format, they were converted to a data format readable by DIAdem using a customized Python program. The measurement data obtained from nanopore measurements showed an inconsistent current baseline owing to the depletion of ions that serve as current carriers and electrode reactions during the measurement. To correct for this baseline trend, data processing software (DIAdem) was used to smooth the median shift value of each of the 3000 measurement data points to obtain the baseline trend, and the difference of that from the original data was taken to correct the baseline to a constant as shown in Figure S4. After correction, the baseline of the measured data became horizontal, and a threshold value was obtained, following which pulse detection was performed. For pulse detection, the pulse detection algorithm provided by SciPy, one of the Python libraries, was used to separate the pulses from the noise floor and to acquire each pulse waveform.

### Frequency Characterization

The applied voltage was 400 mV_pp_, the third-order LPF had a bandwidth of 2 kHz, and the particle driving pressure was 30 Pa. When faster measurements were required for frequency characterization, the third-order LPF was maintained and the bandwidth was increased as necessary. The SNR was low for the measurement of particles with diameters of 200 nm, and only the phase above 30 kHz was detected; however, the current value could not be detected.

Table S1 summarizes the list of particles used in Figure 2, organized by particle size, material, and surface modification. However, the lineup of particle types is not complete because some particles were difficult to obtain.

### Machine Learning

Machine learning was performed using an original code in Python. First, the number of waveforms for each particle was equalized by undersampling to the lowest value of waveform data using the RandomUnderSampler function in the scikit-learn library of Python. Because the maximum and minimum values of the waveform data were not fixed and outliers existed, standardization was performed by scaling the mean to 0 and the variance to 1 using the stats.zsore function in the SciPy library. All feature combinations were searched for using the feature_selection function. The ExhaustiveFeatureSelector function in the Mixtend library was used for the peak height of each waveform and the width of the waveform every 10% when the peak height was set as 100%. For each waveform, the six best combinations with the best learning effect were extracted. The random forest method was used as the learning algorithm, and the ensemble.RandomForestClassifier function of scikit-learn was applied. To verify the generalization performance of the obtained prediction model, *k*-partition cross-validation was performed using the model_selection.StratifiedKFold function and the cross_val_predict function in scikit-learn. In addition, lower frequencies were associated with higher accuracy. The accuracy of machine learning was found to be frequency-dependent: The lower the frequency, the higher the accuracy. This would reflect the fact that the lower the frequency, the higher the difference in magnitude and phase of the current between particle types shown in Figure 2. For viruses, data at 10 kHz, where the measurement was stable, were used for training because the measurement becomes unstable at lower frequencies.

## Supporting Information Available

Applied voltage and measured current waveforms, particle list used to evaluate frequency response, SEM images for the four types of particles in the 1 μm diameter group and three types of particles in the 200 nm diameter group, particle diameter distribution measured using the ac nanopore method, series of detrending processes, with a change in the measured data with respect to time (PDF)
Supporting Movie 1: measured AC signal converted to a DC signal as a root-mean-square value (RMS), yielding a current waveform similar to that obtained by the DC nanopore method (AVI)
Supporting Movie 2: current magnitude and phase changes occuring synchronously, indicating that they originated from a single particle (AVI)

## Supplementary Material

ao3c00628_si_001.pdf

ao3c00628_si_002.avi

ao3c00628_si_003.avi
