# Correction to Nanopore Impedance Spectroscopy Reveals Electrical Properties of Single Nanoparticles for Detecting and Identifying Pathogenic Viruses

**DOI:** 10.1021/acsomega.3c04155

**Published:** 2023-06-30

**Authors:** Kazuki Kitta, Maami Sakamoto, Kei Hayakawa, Akira Nukazuka, Kazuhiko Kano, Takatoki Yamamoto

In eq 2, *R* is modified to *R*_*s*_.
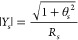


