# Supplementary material for: Nanopore Impedance Spectroscopy Reveals Electrical Properties of Single Nanoparticles for Detecting and Identifying Pathogenic Viruses
Source: ACS Omega. 2023 Apr 6;8(16):14684–93. doi: 10.1021/acsomega.3c00628 (PMC10134219; doi:10.1021/acsomega.3c00628)
Supplement: Supplementary file 1 — ao3c00628_si_001.pdf [file ao3c00628_si_001.pdf]

Nanopore impedance spectroscopy reveals electrical properties of single nanoparticles for detecting and identifying pathogenic viruses

Kazuki Kitta[a‡], *Maami Sakamoto*[a‡], *Kei Hayakawa*[b], *Akira Nukazuka*[b], *Kazuhiko Kano*[b], *and Takatoki Yamamoto*[a*]

[a]Mechanical Engineering, Tokyo Institute of Technology, Ishikawadai 1-314, 2-12-1 Ookayama, Meguro-ku, Tokyo 152-8550, Japan

[b]Material Research and Innovation Division, DENSO CORPORATION, 1-1 Showa-cho, Kariya, Aichi 448-8661, Japan

[*]Correspondence: Takatoki Yamamoto

Email: yamamoto.t.ba@m.titech.ac.jp

[‡]K.K. and M.S. contributed equally to this paper as co-first authors.

Keywords: nanopore, alternating current, lock-in, impedance, virus, machine learning, nanoparticle

a) 1kHz

b) 100kHz

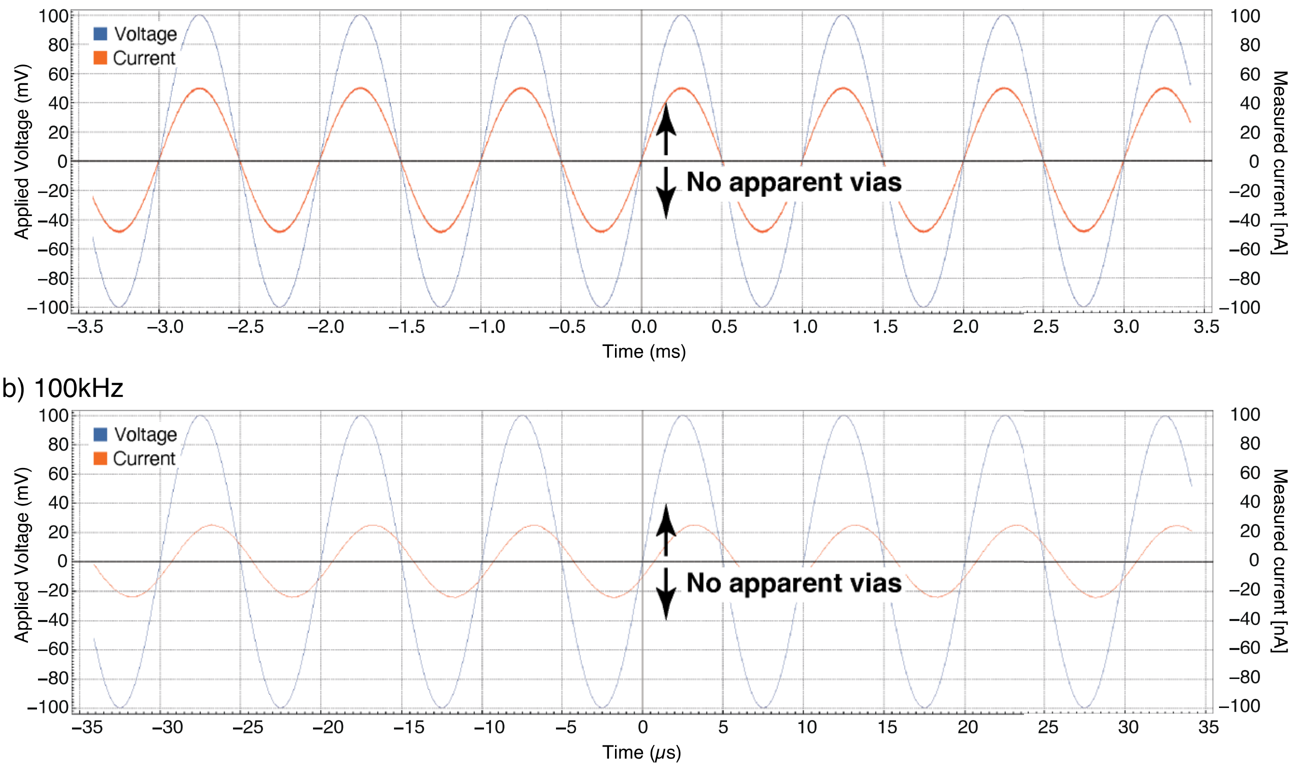

Figure S1: Applied voltage and measured current waveform. No obvious waveform distortion or bias occurred. The measurement conditions included an applied voltage of 0.1 V_{pp} and frequencies of a) 1 kHz and b) 100 kHz.

Table S1: Particle list used to evaluate frequency response.

	Diameter	Material	Surface
**Sample A**	1 μm	Polystyrene	Plain
**Sample B**	1 μm	Polystyrene	COOH
**Sample C**	1 μm	Silica	Plain
**Sample D**	1 μm	Silica	COOH
**Sample E**	200 nm	Polystyrene	Plain
**Sample F**	200 nm	Polystyrene	COOH
**Sample G**	200 nm	Silica	COOH

Two groups of particles of (1 μm and 200 nm in diameter) were used. In the material category, blue represents polystyrene and orange represents silica. In the surface modification category, green represents carboxyl group addition and purple represents unmodified (plain). In the 1-μm diameter group, sample A is polystyrene with an unmodified surface. Sample B is also a polystyrene particle, but its surface is modified with carboxyl groups (COOH). Samples C and D are silica, and sample D is modified with carboxyl groups on the surface. For the 200-nm diameter group, samples E and F are polystyrene particles, unmodified and modified with COOH, respectively. Sample G is a COOH silica particle.

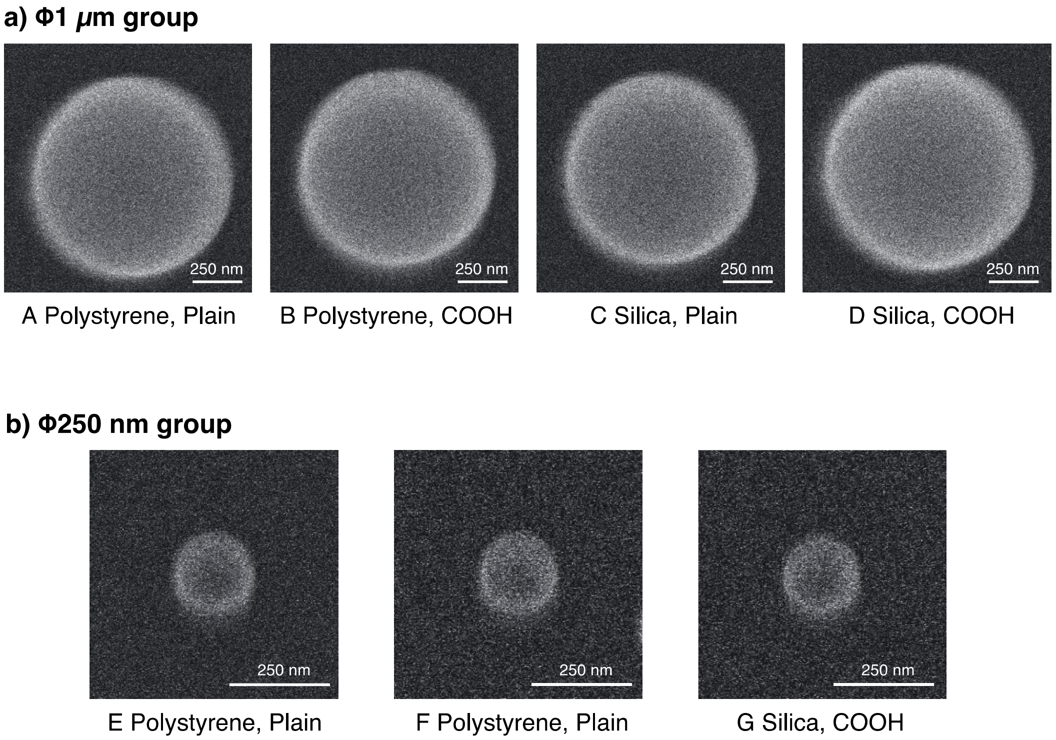

Figure S2: SEM images for the a) four types of particles in the 1-μm diameter group and b) three types of particles in the 200-nm diameter group. The images were taken using JSM-5610 (JEOL Ltd., Tokyo, Japan) at an acceleration voltage of 20 kV. Samples were pre-coated with 2.5 nm osmium using a commercial osmium coater (Neoc-Pro, MEIWAFOSIS Co., Ltd., Tokyo, Japan) to prevent charging up. There is no clear difference in the particle sizes in any of the particle size groups.

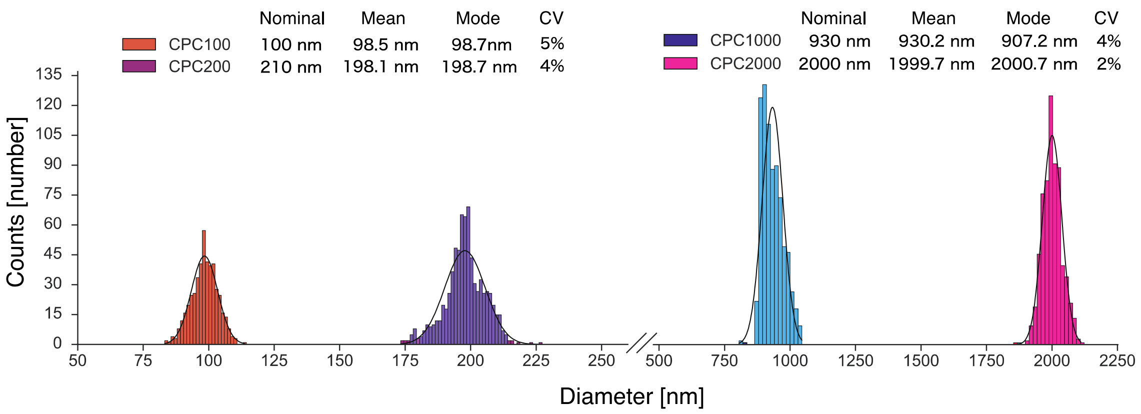

Figure S3: Particle diameter distribution measured using the AC nanopore method with the obtained particle diameter compared with the nominal value of the manufacturer as tracer particles. The group with a diameter range of 100–200 nm and that with a range of several micrometers were measured separately and are displayed as a single graph. For the 100–200 nm group, particles of 100 nm (CPC100) and 210 nm (CPC200) in diameter were measured with the NP200 nanopore chip. For the range of several micrometers, particles of 930 nm (CPC1000) and 2000 nm (CPC2000) in diameter were measured on the NP400 nanopore chip. All these size-standard carboxylated polystyrene particles were purchased from Izon Science Ltd. (Christchurch, New Zealand). The size of the particles was obtained by comparing the magnitude of the current change between the particles with respect to each other. The diameter of the sample particles (d_s) was determined by performing a calibration of $d_s = (I_s/I_{cal})^{1/3} d_{cal}$, using the current change (I_s) of the measured sample particles and the resistance change (I_{cal}) of the calibration particles of known diameter (d_{cal}). Histograms of particle size distribution were plotted after removing outliers that fell within the range of 90% to 10% of the population's particle size (D90/D10). The histogram was created by counting at least 500 particles for each particle size. The black line is the fitting line using the Gaussian distribution, which was used to calculate the mean diameter and coefficient of variation.

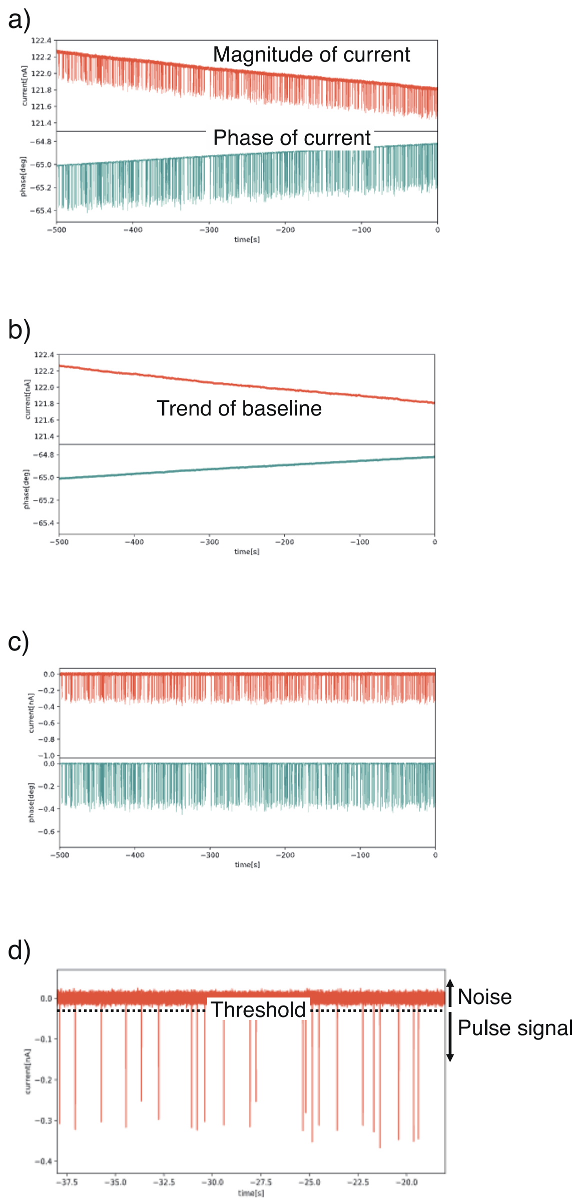

Figure S4: A series of detrending processes is shown. Change in the measured data with respect to time (S4a). The baseline obtained by detrending (S4b) and the corrected measured data (S4c) by taking the difference from the original data so that the baseline remains constant. The noise was separated from the current pulse after correction. A threshold was set to separate the pulse signal from the noise floor (S4d).
